# Neutrophil lymphocyte ratio as a measure of systemic inflammation in prevalent chronic diseases in Asian population

**DOI:** 10.1186/1755-7682-5-2

**Published:** 2012-01-26

**Authors:** Fauzia Imtiaz, Kashif Shafique, Saira Saeed Mirza, Zeenat Ayoob, Priya Vart, Saadiyah Rao

**Affiliations:** 1 Institute of Basic Medical Sciences, Dow International Medical College, Dow University of Health Sciences, Karachi, Pakistan; 2 Institute of Health & Wellbeing, Public Health, University of Glasgow, UK; 3 Centre for Population Health Sciences, University of Edinburgh. UK

**Keywords:** Systemic inflammation, NLR, Co-morbidity, Cancers, Cardiovascular diseases

## Abstract

**Background:**

Preliminary evidence has suggested the role of inflammation in development and prognosis of cardiovascular diseases and cancers. Most of the prognostic studies failed to account for the effects of co-morbid conditions as these might have raised the systemic inflammation. We used neutrophil lymphocyte ratio (NLR) as a measure of systemic inflammation and investigated its association with prevalent chronic conditions.

**Methods:**

Present study is a cross sectional study conducted on population of Karachi, Pakistan. A detailed questionnaire about the demographic details of all subjects was filled and an informed consent obtained for blood sampling. Multinomial regression analyses were carried out to investigate the relationship between NLR and prevalent chronic conditions.

**Results:**

1070 apparently healthy individuals participated in the study. Proportion of individuals with hypertension was higher in middle and highest tertile of NLR as compared to the lowest tertile (18.2% & 16.1% compared to 11.8%). Individuals with hypertension were 43% (RRR = 1.43, 95% CI 0.94-2.20) and 66% (RRR = 1.69, 95% CI 1.09-2.54) more likely to be in the middle and highest tertile of NLR respectively compared to the baseline group. Similarly, individuals with diabetes mellitus were 53% (RRR = 1.53, 95% CI 0.93-2.51) and 65% (RRR = 1.65, 95% CI 1.01-2.71) more likely to be in the middle or highest tertile of NLR as compared to the baseline NLR group.

**Conclusions:**

Systemic inflammation measured by NLR has a significant association with prevalent chronic conditions. Future research is needed to investigate this relationship with longitudinal data to establish the temporal association between these variables.

## Background

Cardiovascular diseases and cancers are leading causes of morbidity and mortality all over the world. The global burden of these diseases has sharply increased in last two decades and continues to augment due to the growth in aging population and health risk behaviours [1,2]. Preliminary evidence has suggested the role of inflammation in development and prognosis of cardiovascular diseases as well as cancers [3-7].

Several studies have explored the relationship between systemic inflammation and cardiovascular mortality [3,5]. Elevated levels of systemic inflammatory markers have been found associated with incidence of cardiovascular diseases [3,5]. Furthermore, many epidemiological studies have highlighted that chronic low grade inflammation is associated with diabetes mellitus [8], hypertension [9], metabolic syndrome [10,11], obesity [12], smoking [13], and other lifestyle habbits [14].

Systemic inflammation can be measured by using a variety of biochemical and haematological markers. Although novel disease specific biomarkers have been identified, most of which are time consuming and expensive. Observational studies have thoroughly investigated the role of C-reactive protein and total leukocyte count in different chronic conditions [6,7,13,15,16].

Low grade inflammation measured by White Blood Cell (WBC) count has also been linked to the traditional risk factors of chronic diseases i.e. smoking, obesity, hypertension and elevated levels of triglycerides [16,17]. Recent evidence indicated that the ratio of sub types of blood cells have a significant prognostic value for cardiovascular disease.

Elevated levels of neutrophil lymphocyte ratio (NLR) were also found associated with poor survival of patients undergoing coronary artery bypass graft[18]. Many cancer survival studies have suggested that NLR is a significant predictor of overall and disease specific survival of patients [19,20]. Whether the systemic inflammation, which has proven to be a significant predictor of survival of cancer patients, is malignancy associated inflammation or is because of the any co-morbid conditions that cancer patients may have is still unclear. We assumed that systemic inflammation might also be raised in commonly prevalent conditions.

Neutrophil lymphocyte ratio could be an important measure of systemic inflammation as it is cost effective, readily available and could be calculated easily. Little is known and published about neutrophil lymphocyte ratio and its relationship with prevalent chronic conditions among general population. Therefore, the current study was conducted to investigate the neutrophil lymphocyte ratio as a measure of systemic inflammation and its relationship, if any, with prevalent chronic diseases.

## Methods

This cross sectional study was conducted in the North of Karachi, the biggest city of Pakistan with a population of 17 millions. We invited 1500 individuals for free medical check up and participation in this study. The study was approved by the independent ethics committee. An information sheet was prepared in English and Urdu to disseminate the objectives of this research study to the participants and written consent was obtained prior to employing a self administered questionnaire.

All willing healthy individuals between the ages of 16 to 75 who accepted the invitation to participate were included in this study. Individuals who visited the hospital due to current illness or follow up of a previous illness were excluded. Furthermore, those who had a smoking habit or used any other addictive substances in last 12 months were also excluded. We used a pre-tested self administered questionnaire which included demographic details, lifestyle habits and history of any known chronic disease and current and past use of medications. We also performed a general physical examination of all participants including height, weight and blood pressure measurements. Height and weight were measured removing the shoes, blood pressure was measured three times with a gap of five minutes and the mean value was calculated. A trained nurse was available at the time of examination, and provided support to participants in filling the questionnaire and in some cases where they were unable to fill the questionnaire themselves, she interviewed the participants. We classified subjects as hypertensive if they had a blood pressure of greater than 140/90 mm/Hg or those who had been diagnosed previously. Diabetes mellitus was defined by a fasting blood glucose level of > 126 mg/dl or a self report of previously diagnosed diabetes mellitus. Existence of other chronic conditions (asthma and arthritis) was ascertained by self reporting.

### Investigations

We obtained blood samples of all individuals who participated in the study to investigate complete blood count, lipid profile and fasting blood glucose. We used Sysmex Pouch counter (An automated machine by S Ejaz uddin & co) for complete blood count. For complete blood count we took 2 ml of blood in a purple top vaccutainer (containing ethylene diamine tetra acetic acid in it in it) and after 5 min mixing on rotator the sample was ran in machine and results were obtained.

### Statistical analysis

We used Stata software version 11 (StataCorp, College Station, TX, USA) to analyze the collected data. We grouped the participants in tertiles of neutrophil lymphocyte ratios. Tertiles were formed to divide the sample into three groups of approximately similar number of participants in each group. Body mass index (BMI) was calculated by using standard formula of weight in kilograms divided by height (metres squared). BMI then categorised into three groups of desirable, overweight and obese using the recommended cut off for Asian population, where a BMI of < 23.5 was considered as desirable, 23.5-27.5 as overweight and > 27.5 as obese [21]. We calculated the mean and standard deviation for continuous variables and frequency for categorical variables. For comparison between groups we used one way Analysis of Variance (ANOVA) with bonferroni adjustments for multiple comparisons of continuous and Kruskal Wallis Test for ordinal variables. We used multinomial logistic regression with lowest category of NLR as baseline and presented relative risk ratios (RRR). In multivariate analysis we adjusted for age, gender, BMI and self reported chronic conditions and taken the first category as reference. All the significance tests were two tailed with a significance level 0.05%.

## Results

### Basic characteristics of study sample

We invited 1500 individuals for this study, 1089 accepted the invitation (response rate 72.6%). Nine individuals were excluded due to current febrile illness while blood count data were missing for 10 individuals so the final analysis included 1070 apparently healthy individuals. We grouped study sample based on neutrophil lymphocyte ratio (NLR) tertiles. Generally, individuals in the highest tertile of NLR were slightly older, more likely to be male, taller, heavier and obese. However, there was no statistically significant difference between three groups of neutrophil lymphocyte ratio. Basic characteristics of study sample have been summarized in table 1.

**Table 1:** Baseline characteristics of study sample based on NLR categories

	Neutrophil Lymphocyte Ratio, Tertiles					
	**0.17-1.51**		**1.52 - < 2.56**		**2.56 - 22.50**	

						
Sample Size	357		361		352	
Mean Age (s.d.)	33.4	(15.1)	34.9	(16.1)	33.0	(14.7)
Gender, % (n)						
Male	34.6	(207)	31.1	(186)	34.4	(206)
Female	31.9	(150)	37.2	(175)	31.0	(146)
Mean Height (s.d.)	172.1	(9.2)	172.1	(8.8)	173.1	(9.0)
Mean Weight (s.d.)	73.1	(14.1)	72.2	(13.9)	75.9	(14.1)
BMI (kg m^-2^), %(n)						
< 23.5 (Desirable)	33.4	(134)	37.2	(149)	29.4	(118)
23.5 - 27.5 (Overweight)	34.2	(143)	33.3	(139)	32.5	(136)
> 27.5 (Obese)	32.5	(79)	28.0	(68)	39.5	(96)
Self reported chronic diseases, %(n)						
Hypertension	11.8	(42)	16.1	(58)	18.2	(64)
Diabetes Mellitus	8.1	(29)	11.9	(43)	12.8	(45)
Asthma	3.1	(11)	5.5	(20)	2.8	(10)
Arthritis	7.0	(25)	8.9	(32)	7.4	(26)
						

### Neutrophil lymphocyte ratio and chronic diseases

Proportion of individuals with hypertension was higher in middle and highest tertile of NLR as compared to the lowest tertile (18.2% & 16.1% compared to 11.8%). Similar trend was observed with diabetes mellitus, while individuals with asthma and arthritis were more likely to be in the middle tertile of NLR.

In a model adjusted for other co-morbid conditions, hypertension and diabetes mellitus were significantly associated with higher NLR, while asthma and arthritis did not show any significant association with NLR. Individuals with hypertension were 43% (RRR = 1.43, 95% CI 0.94-2.20) and 66% (RRR = 1.69, 95% CI 1.09-2.54) more likely to be in the middle and highest tertile of NLR respectively (table 2) compared with baseline group. Similarly individuals with diabetes mellitus were 53% (RRR = 1.53, 95% CI 0.93-2.51) and 65% (RRR = 1.65, 95% CI 1.01-2.71) more likely to be in the middle and highest tertile of NLR as compared with baseline NLR group (table 2). Individuals with asthma and arthritis were 84% and 29% more likely to be in middle tertile of NLR respectively, however this association was not statistically significant (table 2).

**Table 2:** Relative risk ratio of higher neutrophil lymphocyte ratio (compared to NLR 0.17-1.51)

	**Neutrophil Lymphocyte Ratio (Tertiles)**				
		
	**1.52-2.56**		**2.57-22.5**		
				
**Characteristics**	Relative Risk Ratio*	**p value**	Relative Risk Ratio*	**p value**	
	
	**(95% CI)**		**(95% CI)**		
**Self reported chronic condition**					
Hypertension					
No	1		1		
Yes	1.43 (0.94-2.20)	0.09	1.66 (1.09-2.54)	0.01	
Diabetes					
No	1		1		
Yes	1.53 (0.93-2.51)	0.09	1.65 (1.01-2.71)	0.04	
Asthma					
No	1		1		
Yes	1.84 (0.87-3.90)	0.11	0.92 (0.39-2.19)	0.85	
Arthritis					
No	1		1		
Yes	1.29 (0.70-2.22)	0.36	1.06 (0.60-1.87)	0.84	

* Relative risk ratio presented after mutual adjustments of all the covariates presented in the table

**Figure 1:**
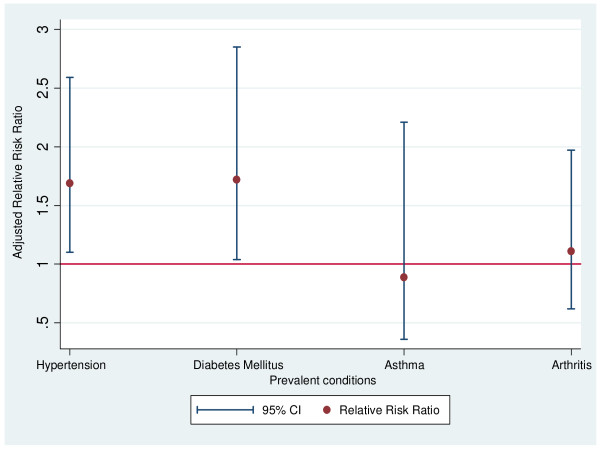
**Adjusted relative risk ratio of highest tertile from a multivariate model**. Relative risk ratio was adjusted for age, sex, body mass index and self reported conditions.

We further adjusted for the effects of age, gender and body mass index. In this final multivariate model, NLR has significant association with hypertension and diabetes mellitus (Figure 1). Individuals with asthma and arthritis were more likely to be in the middle group of NLR tertiles but the association was not significant. Age, gender and body mass index did not have any significant association with NLR in final multivariate model (table 3). Furthermore, analysis was also repeated by excluding hypertensive participants (n = 17) who were taking other drugs including Statins and/or aspirin but overall relationship remains consistent and hypertensive individuals were 56% (RRR 1.56, 95% CI 1.14-2.45, p-value 0.03) more likely to be in the highest tertile of NLR. Overall relationship between diabetes mellitus and NLR also remained consistent and significantly differed after these exclusions. We also categorised the NLR into quintiles and taken the first category as reference but this did not materially altered the above mentioned associations.

**Table 3:** Relative risk ratio of higher neutrophil lymphocyte ratio (compared to NLR 0.17-1.51)

	Neutrophil Lymphocyte Ratio (Tertiles)*			
	
	1.52-2.56		2.57-22.5	
				
Characteristics	Relative Risk Ratio	p value	Relative Risk Ratio	p value
	(95% CI)		(95% CI)	
**Age at screening (years)**				
Age< 40	1	_	1	_
Age 40-49	1.38 (0.87-2.18)	0.2	1.44 (0.91-2.25)	0.12
Age 50-59	1.19 (0.75-1.89)	0.46	0.66 (0.39-1.11)	0.12
Age 60-75	1.17 (0.69-1.99)	0.56	1.09 (0.64-1.56)	0.75
**Gender**				
Female	1	_	1	_
Male	0.78 (0.57-1.04)	0.1	1.02 (0.75-1.38)	0.9
**Body mass index (kg/m^2^)**				
Desirable, bmi < 23	1	_	1	_
Overweight, bmi 23-27.5	0.91 (0.65-1.27)	0.56	1.13 (0.80-1.60)	0.47
Obese, bmi > 27.5	0.78 (0.52-1.16)	0.22	1.42 (0.96-2.11)	0.07
**Self reported chronic condition**				
Hypertension				
No	1		1	
Yes	1.43 (0.93 - 2.20)	0.1	1.69 (1.10 - 2.59)	0.02
Diabetes				
No	1		1	
Yes	1.38 (0.83-2.29)	0.22	1.72 (1.04-2.85)	0.03
Asthma				
No	1		1	
Yes	1.53 (0.70-3.34)	0.28	0.89 (0.36-2.21)	0.8
Arthritis				
No	1		1	
Yes	1.27 (0.73-2.21)	0.4	1.11 (0.62-1.97)	0.73

* Relative risk ratio presented after mutual adjustments of all the covariates presented in the table

## Discussion

In this study, we found a significant association between neutrophil lymphocyte ratio (NLR) and likelihood of having hypertension and diabetes mellitus. However, we did not observe a significant relationship between NLR and asthma or arthritis. These relationships remain unchanged even after adjusting for age, gender and obesity. Our study confirms that co-morbid conditions are associated with higher level of systemic inflammation as measured by NLR. However, we could not explore whether the low grade inflammation in the body was a cause of the co-morbid conditions or an effect. Higher level of white blood cells has been previously linked with incident hypertension among different population based studies [9]. Similarly, systemic inflammation has also been reported as a significant factor for metabolic syndrome including obesity and diabetes mellitus [11].

Mechanism underlying these associations between systemic inflammation and prevalent conditions remains to be elucidated. One potential explanation is that, cellular response of blood components might be mediated through the endothelial dysfunction. Inflammation modifies endothelial function and an inability of the endothelium to produce nitric oxide and prostacyclin can result in the depletion of vasodilator, antithrombotic and anti-atherogenic properties of the vascular endothelium[9]. In addition, stimulated leukocytes alter rheological properties with an increased capacity to adhere to vascular endothelium and may result in capillary leukocytosis and subsequent increased vascular resistance[9].

Furthermore, several studies have suggested that chronic, low grade; subclinical inflammation play a major role in development of insulin resistance which may then proceed to development of clinically overt diabetes mellitus [8,22]. Risk factors of diabetes mellitus, such as obesity, smoking and physical inactivity are associated with chronic low grade inflammation. So this also explains our findings of a significant relationship between systemic inflammation and diabetes mellitus.

We did not find any association of inflammatory markers with asthma and arthritis in this study; this may be explained by intake of anti-inflammatory medications among these individuals. Unlike, hypertension and diabetes mellitus, both asthma and arthritis can cause significant pain and discomfort to individuals which lead them towards immediate remedy of the symptoms. In this case, role of inflammation and NLR might have been masked by the intake of anti-inflammatory and pain killer drugs.

To our knowledge this is the first study which investigated the role of neutrophil lymphocyte ratio as measure of systemic inflammation in relation to prevalent chronic conditions in general population. We have a reasonable sample and fair number of cases with each disease to explore any possible relationship between NLR and co-morbid conditions.

There are few limitations of this study which are worth to be reported. First, we used a cross sectional design for this study, which is not the best design to investigate any causal relationships, however this study does raise some questions whether the systemic inflammation is an effect of chronic condition or is it a cause of it. Future research with prospective design and multiple measurements of NLR could provide more robust evidence on role of NLR in relation to co-morbid conditions. Second, it is possible that the relationship between elevated NLR and co-morbid conditions might have confounded by some unmeasured co-variates. However, as we selected apparently healthy individuals from the population, this seems unlikely. Finally some element of bias can not be excluded as individuals with high level of systemic inflammation are more likely to visit a doctor and this sample might not be a true representative of the whole population. However, our random invitations might have eliminated some of the effects caused by chance.

Despite the limitations of the study, we observed that individuals with prevalent chronic conditions are significantly more likely to have higher level of systemic inflammation and NLR seems a reasonable measure to detect this condition..

## Conclusions

Systemic inflammation measured by NLR has significant association with prevalent chronic conditions. Future research needs to investigate the relationship with longitudinal data to establish the temporal relationship between these.

## Competing interests

The authors declare that they have no competing interests.

## References

[B1] Jemal A Bray F Center MM Ferlay J Ward E Forman D Global cancer statistics CA Cancer J Clin 2011 61 69 90 10.3322/caac.20107 21296855

[B2] Sanderson JE Mayosi B Yusuf S Reddy S Hu S Chen Z Global burden of cardiovascular disease Heart 2007 93 1175 10.1136/hrt.2007.131060 17890692 PMC2000927

[B3] Folsom AR Wu KK Rosamond WD Sharrett AR Chambless LE Prospective study of hemostatic factors and incidence of coronary heart disease: the Atherosclerosis Risk in Communities (ARIC) Study Circulation 1997 96 1102 1108 9286936 10.1161/01.cir.96.4.1102

[B4] Folsom AR Rosamond WD Shahar E Cooper LS Aleksic N Nieto FJ Prospective study of markers of hemostatic function with risk of ischemic stroke. The Atherosclerosis Risk in Communities (ARIC) Study Investigators Circulation 1999 100 736 742 10449696 10.1161/01.cir.100.7.736

[B5] Folsom AR Aleksic N Catellier D Juneja HS Wu KK C-reactive protein and incident coronary heart disease in the Atherosclerosis Risk In Communities (ARIC) study Am Heart J 2002 144 233 238 10.1067/mhj.2002.124054 12177639

[B6] Lee S Choe JW Kim HK Sung J High-Sensitivity C-Reactive Protein and Cancer J Epidemiol 2011 10.2188/jea.JE20100128 PMC3899404 21368452

[B7] Saito K Kihara K Role of C-reactive protein as a biomarker for renal cell carcinoma Expert Rev Anticancer Ther 2010 10 1979 1989 10.1586/era.10.192 21110763

[B8] Pitsavos C Tampourlou M Panagiotakos DB Skoumas Y Chrysohoou C Nomikos T Association Between Low-Grade Systemic Inflammation and Type 2 Diabetes Mellitus Among Men and Women from the ATTICA Study Rev Diabet Stud 2007 4 98 104 10.1900/RDS.2007.4.98 17823694 PMC2036265

[B9] Nakanishi N Sato M Shirai K Suzuki K Tatara K White blood cell count as a risk factor for hypertension; a study of Japanese male office workers J Hypertens 2002 20 851 857 10.1097/00004872-200205000-00018 12011644

[B10] Bell DS O'Keefe JH White cell count, mortality, and metabolic syndrome in the Baltimore longitudinal study of aging J Am Coll Cardiol 2007 50 1810 1811 17964048 10.1016/j.jacc.2007.05.052

[B11] Marsland AL McCaffery JM Muldoon MF Manuck SB Systemic inflammation and the metabolic syndrome among middle-aged community volunteers Metabolism 2010 59 1801 1808 10.1016/j.metabol.2010.05.015 20619428 PMC2955187

[B12] Brooks GC Blaha MJ Blumenthal RS Relation of C-reactive protein to abdominal adiposity Am J Cardiol 2010 106 56 61 10.1016/j.amjcard.2010.02.017 20609648

[B13] Yasue H Hirai N Mizuno Y Harada E Itoh T Yoshimura M Low-grade inflammation, thrombogenicity, and atherogenic lipid profile in cigarette smokers Circ J 2006 70 8 13 10.1253/circj.70.8 16377917

[B14] Pirkola J Vaarasmaki M Ala-Korpela M Bloigu A Canoy D Hartikainen AL Low-grade, systemic inflammation in adolescents: association with early-life factors, gender, and lifestyle Am J Epidemiol 2010 171 72 82 10.1093/aje/kwp320 19917553

[B15] Bovill EG Bild DE Heiss G Kuller LH Lee MH Rock R White blood cell counts in persons aged 65 years or more from the Cardiovascular Health Study. Correlations with baseline clinical and demographic characteristics Am J Epidemiol 1996 143 1107 1115 8633599 10.1093/oxfordjournals.aje.a008687

[B16] Freedman DS Joesoef MR Barboriak JJ Stallone DD Byers T Correlates of leukocyte counts in men Ann Epidemiol 1996 6 74 82 10.1016/1047-2797(95)00091-7 8680629

[B17] Facchini F Hollenbeck CB Chen YN Chen YD Reaven GM Demonstration of a relationship between white blood cell count, insulin resistance, and several risk factors for coronary heart disease in wome J Intern Med 1992 232 267 272 10.1111/j.1365-2796.1992.tb00582.x 1402624

[B18] Gibson PH Croal BL Cuthbertson BH Small GR Ifezulike AI Gibson G Preoperative neutrophil-lymphocyte ratio and outcome from coronary artery bypass grafting Am Heart J 2007 154 995 1002 10.1016/j.ahj.2007.06.043 17967611

[B19] Walsh SR Cook EJ Goulder F Justin TA Keeling NJ Neutrophil-lymphocyte ratio as a prognostic factor in colorectal cancer J Surg Oncol 2005 91 181 184 10.1002/jso.20329 16118772

[B20] Sarraf KM Belcher E Raevsky E Nicholson AG Goldstraw P Lim E Neutrophil/lymphocyte ratio and its association with survival after complete resection in non-small cell lung cancer J Thorac Cardiovasc Surg 2009 137 425 428 10.1016/j.jtcvs.2008.05.046 19185164

[B21] World Health Organization, Western Pacific Region. The International Associationfor the Study of Obesity and the International Obesity Task Force. The Asia-Pacific perspective: redefining obesity and its treatment. Sydney, Australia Health Communications Australia Pty Limited; 2000 2010 Available: www diabetes com au/pdf/obesity_report pdf20331414

[B22] Friedman GD Tekawa I Grimm RH Manolio T Shannon SG Sidney S The leucocyte count: correlates and relationship to coronary risk factors: the CARDIA study Int J Epidemiol 1990 19 889 893 10.1093/ije/19.4.889 2084017

